# A Mathematical Description of the Bone Marrow Dynamics during CAR T-Cell Therapy in B-Cell Childhood Acute Lymphoblastic Leukemia

**DOI:** 10.3390/ijms22126371

**Published:** 2021-06-14

**Authors:** Álvaro Martínez-Rubio, Salvador Chulián, Cristina Blázquez Goñi, Manuel Ramírez Orellana, Antonio Pérez Martínez, Alfonso Navarro-Zapata, Cristina Ferreras, Victor M. Pérez-García, María Rosa

**Affiliations:** 1 Department of Mathematics, Universidad de Cádiz, Puerto Real, 11510 Cádiz, Spain; Salvador.chulian@uca.es (S.C.); maria.rosa@uca.es (M.R.); 2 Biomedical Research and Innovation Institute of Cádiz (INiBICA), Hospital Universitario Puerta del Mar, 11009 Cádiz, Spain; cblazquezgoni@yahoo.es; 3 Department of Pediatric Hematology and Oncology, Hospital de Jerez, 11407 Cádiz, Spain; 4 Department of Paediatric Haematology and Oncology, Instituto Investigación Sanitaria La Princesa, Hospital Infantil Universitario Niño Jesús, 28006 Madrid, Spain; manuel.ramirez@salud.madrid.org; 5 Translational Research in Pediatric Oncology, Hematopoietic Transplantation and Cell Therapy, IdiPAZ, Hospital Universitario La Paz, 28046 Madrid, Spain; antonioperezmartinez@yahoo.es (A.P.M.); alfonso.navarro.zapata@idipaz.es (A.N.-Z.); cristina.idipaz@gmail.com (C.F.); 6 Pediatric Hemato-Oncology Department, Hospital Universitario La Paz, 28046 Madrid, Spain; 7 Mathematical Oncology Laboratory (MOLAB), Instituto de Matemática Aplicada a la Ciencia y la Ingeniería, Universidad de Castilla-La Mancha, 13005 Ciudad Real, Spain; victor.perezgarcia@uclm.es; 8 Departamento de Matemáticas, Escuela Técnica Superior de Ingenieros Industriales, Universidad de Castilla-La Mancha, 13005 Ciudad Real, Spain

**Keywords:** CAR T, mathematical model, acute lymphoblastic leukemia, B cell, bone marrow

## Abstract

Chimeric Antigen Receptor (CAR) T-cell therapy has demonstrated high rates of response in recurrent B-cell Acute Lymphoblastic Leukemia in children and young adults. Despite this success, a fraction of patients’ experience relapse after treatment. Relapse is often preceded by recovery of healthy B cells, which suggests loss or dysfunction of CAR T-cells in bone marrow. This site is harder to access, and thus is not monitored as frequently as peripheral blood. Understanding the interplay between B cells, leukemic cells, and CAR T-cells in bone marrow is paramount in ascertaining the causes of lack of response. In this paper, we put forward a mathematical model representing the interaction between constantly renewing B cells, CAR T-cells, and leukemic cells in the bone marrow. Our model accounts for the maturation dynamics of B cells and incorporates effector and memory CAR T-cells. The model provides a plausible description of the dynamics of the various cellular compartments in bone marrow after CAR T infusion. After exploration of the parameter space, we found that the dynamics of CAR T product and disease were independent of the dose injected, initial B-cell load, and leukemia burden. We also show theoretically the importance of CAR T product attributes in determining therapy outcome, and have studied a variety of possible response scenarios, including second dosage schemes. We conclude by setting out ideas for the refinement of the model.

## 1. Introduction

Chimeric antigen receptor (CAR) T-cell therapy is an immunotherapy technique consisting of the genetic modification of T-cells to allow them to recognize specific tumor antigens. The CAR T product is built by obtaining T-cells from the patient’s blood, and engineering and expanding them in the laboratory. The product is then re-infused into the patient for therapeutic purposes [1]. This is regarded as one of the most promising therapeutic advances in the fight against cancer, and is now integrated into standard care for some hematological malignancies [2,3]. The most successful instance of CAR T therapy is its application to B-cell malignancies, especially when aimed at T-cells bearing the B-cell common antigen CD19. This marker is widely expressed in B cells but is absent from other cell types [4], making it an ideal target for immunotherapy. High response rates have been reported in B-cell Acute Lymphoblastic Leukemias and in Diffuse Large B-cell Lymphomas [5,6,7], leading to the approval of several CAR T products by different regulatory agencies [8,9].

This paper focuses on the use of CAR T in B-cell childhood Acute Lymphoblastic Leukemia (ALL). This cancer comprises 80% of pediatric leukemias, which account for 25% of all pediatric malignancies [10]. The rate of cure of B-cell ALL has been increasing steadily thanks to improvements in chemotherapy regimes, with around 80% of children achieving complete remission [11]. Prognosis is much worse for patients who experience relapse. This set of patients usually receives additional chemotherapy cycles and possibly hematopoietic progenitor transplants, which are frequently ineffective [12]. It is in this group of highly treated patients that a good rate of response to CAR T therapy has been observed [13]. Clinical trials have shown that CAR T-cells are able to expand in vivo, eliminate leukemia burden, and persist in the patient for as long as two years [6,14,15,16].

Despite this success, 30 to 50% of patients still experience long term relapse [17]. Moreover, 20% of patients fail to achieve remission after CAR T infusion. Some fraction of these relapses are caused by what has been termed “antigenic escape,” meaning that leukemic cells lose expression of the CD19 antigen and thus avoid the targeted action of CAR T-cells. CD19^+^ relapse, on the other hand, is related to a lack of expansion or persistence of the product [3]. Clinical trials and associated research have found conflicting evidence regarding the relationship between dose, leukemia burden, and response. It has been pointed out that the reasons for treatment failure may be drug-intrinsic, probably related to characteristics of the patient’s T-cells before extraction and manufacturing [18,19].

CAR T therapy presents a number of treatment-associated toxicities, the most important of which is the so-called cytokine release syndrome (CRS). This condition is related to the fast action of T-cells against CD19^+^ cells, and therefore linked to the level of CAR expansion and leukemia burden. Neurotoxicity is another, less elucidated side effect. Finally, B-cell aplasia (BCA) is a kind of on-target off-tumor toxicity due to the fact that healthy B cells also express CD19 [20,21]. In fact, BCA in peripheral blood is used as a surrogate marker for CAR T persistence, and loss of BCA is associated with a higher probability of disease recurrence [22,23]. This is because new B cells are continuously being generated in the bone marrow, providing an endless source of stimulation for the CAR T-cell population and acting as an endogenous vaccine [24]. In this regard, it is important to note that most data about the outcome of the therapy, such as absolute leukocyte count, cytokine levels, or B cell and CAR T number, come from peripheral blood samples. However, leukemia is a disease of the bone marrow, where blood cells are produced. From that perspective, peripheral blood can be thought of only as a surrogate marker, since what is observed in blood has probably occurred before or is linked to what has previously occurred in bone marrow. Obtaining aspirates or bone marrow biopsies are more invasive procedures and are not carried out as often as peripheral blood extraction. Thus, data on relevanT-cellular components in bone marrow dynamics are scarce. Recent studies related to clinical trials have nonetheless recognized the importance of tracking bone-marrow dynamics [24].

This is where mathematical modeling comes into play. Mathematical models have a long history of application been applied in biology, medicine, and oncology. Like animal models, mathematical models offer a simplified representation of a system, providing a tool to explore causal relations and mechanisms. As a recent therapeutic breakthrough, CAR T therapy is starting to lend itself to a mathematical characterization [25]. While there are some studies involving solid tumors [26,27], most focus on hematological malignancies, given the success obtained in this group of cancers. There are general models of leukemia and lymphoma [28,29,30,31] and more specific applications in B-cell lymphoma [32], B-cell chronic lymphoblastic leukemia [33], T-cell ALL [34], and B-cell ALL [35,36].

By integrating mathematical models of B-cell development and T-cell response, the aim is to describe the interaction between cancerous cells, healthy B cells and CAR T-cells, with the purpose of reproducing and shedding light on the clinical features typically observed in clinical trials. This can also provide a platform for testing hypotheses related to the success or failure of the therapy and for discussing matters related to the response of T-cells to antigen stimulation.

## 2. Mathematical Model

### 2.1. Main Elements of the Model

**Figure 1:**
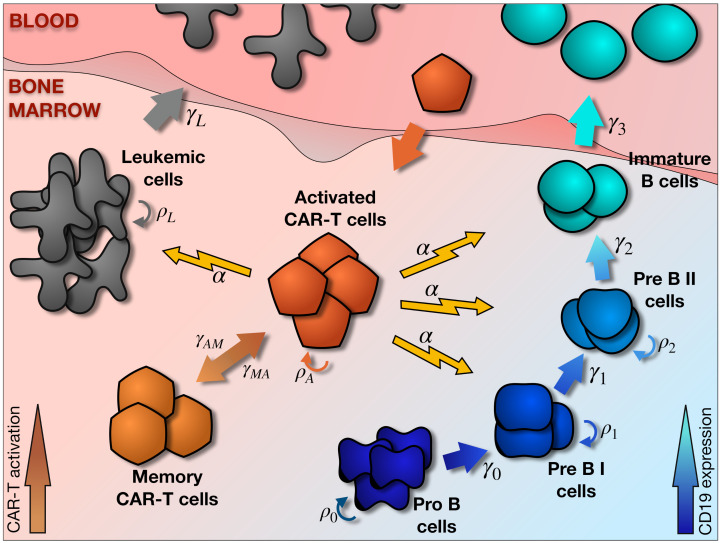
B cells progressively express CD19 as they become more mature. Four maturation stages were considered, namely Pro B, Pre BI, Pre BII, and immature B cells. These cell types were assumed to proliferate with rate $\rho_{i}$ (i=0,…,2) and progress with rates $\gamma_{j}$ (j=0,…,3), except for immature B cells that no longer proliferate, leaving bone marrow with an exit rate $\gamma_{3}$. Leukemic cells originate from a B-cell precursor and proliferate with rate $\rho_{L}$, invading the bone marrow and eventually migrating to peripheral blood with an exit rate $\gamma_{L}$. CAR T-cells are assumed to travel to bone marrow from blood and proliferate with rate $\rho_{C}$ upon encounters with CD19-expressing cells. They carry out effector functions on CD19 expressing cells with killing capacity α. Pro-B cells are not affected by this action since they do not express CD19. Part of the activated CAR T pool becomes long-lived memory cells with rate $\gamma_{AM}$. These cells can regain effector function upon repeated exposure to CD19, with reactivation rate $\gamma_{MA}$.

A schematic representation of the compartments and mechanisms accounted for in our mathematical model is shown in Figure 1. There are three main elements. The first is the production of B cells, a sequential process in which cells transit from Pro-B cells (B-cell progenitors that do not yet express the characteristic CD19 surface marker) to immature B cells which exit the bone marrow to complete maturation elsewhere. There are two intermediate steps which are usually called Pre-BI and Pre-BII [37]. This follows the typical compartmentalization of B-cell development [38]. The second element of the model is the leukemic cells. They arise from one of the precursor stages of B-cell development, either Pre-BI or Pre-BII, and exit the normal maturation route. They then proliferate with the only restriction of available bone marrow space. Some of these leukemic cells can exit the bone marrow and manifest in peripheral blood. Finally, CAR T-cells are infused in peripheral blood and then travel to the bone marrow in order to engage with CD19^+^ cells. They proliferate on encountering them and differentiate into effector cells, inducing the death of antigen-bearing cells and dying shortly afterwards. Part of the expanded population differentiates into long-lived memory cells that retain the ability to proliferate again upon repeated exposure to the antigen [39].

### 2.2. Hematopoietic and Leukemic Compartments

We described healthy bone marrow by means of compartments $B_{i}=B_{i}\left(t\right)$, where i=0,1,2,3 represents different stages of maturation of B lymphocytes. $B_{0}$ are the B-cell progenitors or Pro-B cells, which do not yet express the B-cell antigen CD19; $B_{1}$ are early or Pre-BI cells; $B_{2}$ are intermediate or Pre-BII cells; and $B_{3}$ are late or immature B cells, ready to enter the blood and complete maturation elsewhere. These three last stages already express the marker CD19. The leukemic clone is given by L(t). The mathematical model for the disease without treatment reads:

(1) $$\begin{array}{cc} \frac{dB_{0}}{dt} & =s_{B}\rho_{0}B_{0}-\gamma_{0}B_{0}, \end{array}$$

(2) $$\begin{array}{cc} \frac{dB_{1}}{dt} & =s_{B}\rho_{1}B_{1}+\gamma_{0}B_{0}-\gamma_{1}B_{1}, \end{array}$$

(3) $$\begin{array}{cc} \frac{dB_{2}}{dt} & =s_{B}\rho_{2}B_{2}+\gamma_{1}B_{1}-\gamma_{2}B_{2}, \end{array}$$

(4) $$\begin{array}{cc} \frac{dB_{3}}{dt} & =\gamma_{2}B_{2}-\gamma_{3}B_{3}, \end{array}$$

(5) $$\begin{array}{cc} \frac{dL}{dt} & =s_{L}\rho_{L}L\left(1-\frac{L}{L_{\max}}\right)-\gamma_{L}L, \end{array}$$

with

(6) $$\begin{array}{cc} s_{B}\left(t\right) & =\frac{1}{1+k\left(\sum_{i=0}^{3}B_{i}+L\right)}, \end{array}$$

(7) $$\begin{array}{cc} s_{L}\left(t\right) & =\frac{1}{1+k\left(\sum_{i=0}^{3}B_{i}\right)}. \end{array}$$

This paper looks at two main biological processes within each B-cell compartment: proliferation $\rho_{i}$ and maturation $\gamma_{j}$. In previous works, we followed the hypothesis that the regulation of cell production occurs through a negative feedback signal affecting proliferation $s_{B}\left(t\right)$, where *k* represents the intensity of the signal [40]. This compartmentalized structure with proliferation, maturation, and regulatory feedback has already been used in mathematical models of B-cell hematopoiesis [41]. In this case, the leukemic clone intervenes in this process by inhibiting the growth of healthy B cells. Leukemic cells, however, can avoid self-inhibition and proliferate ($\rho_{L}$) with the restriction of bone marrow space $L_{max}$, invading the bone marrow and leading to a shortage of healthy immune cells [42] (Note that saturation is rarely achieved due to therapeutic action). A different signaling function $s_{L}\left(t\right)$ was therefore specified, without the clone. We assumed that leukemic cells and B-cells both employ the same processes (e.g., cytokine secretion) for this inhibition, and that the larger influence of leukemia is due to their exaggerated growth rather than to different intensity of inhibition. We thus specified the same intensity *k* for both cell types. Finally, we assumed that the invasive character of the clone allows it to exit the bone marrow with rate $\gamma_{L}$ and thus be found in blood, which allows it to eventually settle somewhere else [10].

### 2.3. CAR T-Cell Compartment

Several studies have incorporated CAR T-cells in the form of a compartment that expands and acts on B cells and cancer cells [26,32,34,35,36]. However, clinical evidence suggests that a better description of the dynamics can be achieved by considering two subpopulations [22,23,24]. This aligns with previous models of immune T-cell response, which considered three compartments for the T-cell populations: naïve, activated or effector, and memory cells [43]. Some models of CAR T response have also included this feature [28,31,33]. The process of T-cell response can be summarized as follows: Naïve T-cells enter the system and activate after encountering the corresponding antigen. Activated T-cells then undergo fast expansion and carry out effector functions on the antigen-bearing cells. The majority of this expanded pool die shortly after, but part of these cells become memory T-cells that can reactivate if they encounter the antigen again in the future. The intricacies of this dual differentiation into effector and memory are not yet fully understood [44,45]. The mathematical representation of de Boer et. al. [46], who fitted data of T-cell response to lymphocytic choriomeningitis virus, has been followed here. This infection induces a persistent immune response and therefore can be helpful in simulating CAR T response. In our case, we assumed naïve cells to come from a single infusion and become activated over a short period of time, thereby omitting the corresponding equation. We denote activated cells by $C_{A}\left(t\right)$, and memory cells by $C_{M}\left(t\right)$. Adding these equations to model (1)–(7) yields:

(8) $$\begin{array}{cc} \frac{dB_{0}}{dt} & =s_{H}\rho_{0}B_{0}-\gamma_{0}B_{0}, \end{array}$$

(9) $$\begin{array}{cc} \frac{dB_{1}}{dt} & =s_{H}\rho_{1}B_{1}+\gamma_{0}B_{0}-\gamma_{1}B_{1}-\alpha B_{1}C_{A}, \end{array}$$

(10) $$\begin{array}{cc} \frac{dB_{2}}{dt} & =s_{H}\rho_{2}B_{2}+\gamma_{1}B_{1}-\gamma_{2}B_{2}-\alpha B_{2}C_{A}, \end{array}$$

(11) $$\begin{array}{cc} \frac{dB_{3}}{dt} & =\gamma_{2}B_{2}-\gamma_{3}B_{3}-\alpha B_{3}C_{A}, \end{array}$$

(12) $$\begin{array}{cc} \frac{dL}{dt} & =s_{L}\rho_{L}L\left(1-\frac{L}{L_{\max}}\right)-\gamma_{L}L-\alpha LC_{A}, \end{array}$$

(13) $$\begin{array}{cc} \frac{dC_{A}}{dt} & =F\left(t\right)\left(\rho_{C}C_{A}+\gamma_{MA}C_{M}\right)-\frac{1}{\tau_{A}}C_{A}-\left(1-F\left(t\right)\right)\gamma_{AM}C_{A}, \end{array}$$

(14) $$\begin{array}{cc} \frac{dC_{M}}{dt} & =\left(1-F\left(t\right)\right)\gamma_{AM}C_{A}-\frac{1}{\tau_{M}}C_{M}-F\left(t\right)\gamma_{MA}C_{M}, \end{array}$$

with

(15) $$F\left(t\right)=\frac{\left(\sum_{i=1}^{3}B_{i}+L\right)}{h+\left(\sum_{i=1}^{3}B_{i}+L\right)}.$$

Activated cells $C_{A}\left(t\right)$ proliferate with rate $\rho_{C}$ and become memory cells $C_{M}\left(t\right)$ with rate $\gamma_{AM}$. These processes are modulated by the activation function F(t). This is a continuous function of the level of antigen, and takes values on the interval [0,1]. It has been shown previously that a Michaelis–Menten function provides an appropriate functional form [47], saturating for extended antigen exposure. The Michaelis–Menten constant *h* represents the number of CD19 expressing cells for which activation is half-maximal; we therefore renamed this parameter to activation threshold. This saturating function allowed us to explicitly consider in the model the fact that there is a limit for proliferation during activation, which is not the case in predator–prey models: T-cells commit to a clonal expansion of a given size upon activation of the first parent T-cell [44,48]. Activated cells can also come from memory cells with rate $\gamma_{MA}$, a process that again depends on the activation function. When the amount of antigen decreases, proliferation and activation decrease and transition to memory is initiated, thus the term (1−F(t)). With respect to lifetimes, activated cells die with a characteristic time $\tau_{A}$ of the order of days. Memory cells come from activated cells and have a longer lifetime $\tau_{M}$. They become activated again when the activation function F(t) grows. Finally, the equations for the CD19^+^ compartments, namely $B_{1}\left(t\right)$, $B_{2}\left(t\right)$, $B_{3}\left(t\right)$, and L(t), include an additional mass action term representing elimination by activated CAR T-cells $C_{A}\left(t\right)$, with killing capacity α [49].

To sum up, the major assumptions of the model are: (i) the B-cell developmental dynamics specified as a proliferation and maturation process with inhibitory feedback; (ii) the leukemic clone growing without self-inhibition; and (iii) the CAR T population consisting of activated and memory cells controlled by a saturating activation function.

### 2.4. Parameter Estimation

We studied proliferation and differentiation parameters in a previous work [40], where the evolution of the three CD19^+^ cellular compartments ($B_{1}$, $B_{2}$, and $B_{3}$) was compared to clinical and literature data to obtain characteristic parameter values. We followed the reasoning in that work to provide values for the Pro-B compartment and for the leukemic cell population. A more detailed explanation and references to experimental works can be found in the Supplementary Information. For the CAR T-cell compartment, typical parameter values can be obtained from studies of Tisagenlecleucel kinetics, the FDA-approved CAR T product for B-cell ALL [23,24]. There, the authors fitted patient data to a mixed-effects model and provided values for proliferation rate $\rho_{C}$, memory transition rate $\gamma_{AM}$, and both activated and memory characteristic lifetimes $\tau_{A}$ and $\tau_{M}$. The estimation of the rest of parameters is detailed in the Supplementary Information. All parameter values and their meanings are listed in Table 1.

To simulate Equations (8)–(14), we also had to set the initial conditions for the different compartments. For B cells, we started from the homeostatic concentrations that can be obtained by simulating only Equations (1)–(4). Alternatively, these proportions can be found in ref. [40]. The total B-cell population in these conditions would be around $3\times 10^{10}$cells. CAR T-cell patients, however, undergo a preparatory chemotherapy to remove the host’s lymphocytes (lymphodepleting chemotherapy), so the B-cell population should be substantially smaller. We select as the initial state 1% of that amount leading to a B-cell population of around $3\times 10^{8}$ cells. With respect to the leukemic clone, clinical trials of CAR T therapy in B-cell ALL report leukemia burden ranging from less than 1% to almost 100% of bone marrow mononucleated cells [15,16]. We therefore selected an initial leukemia burden in the range $10^{10}$–$10^{12}$. Finally, CAR T dose was carefully selected in early clinical trials and lies approximately in the range 0.1 to $5\times 10^{6}$ cells per kg [6,14,15,16]. After infusion, CAR T-cells in blood decrease sharply due to their distribution to the tissues [22]. We can assume that a substantial part of the product is transferred to the bone marrow, where the majority of the CD19 antigen is concentrated. Taking into account pediatric weights, we selected the range of $10^{7}$–$10^{8}$ cells injected.

### 2.5. Computational Details

The system of Equations (8)–(14) was solved numerically with the scientific software package Matlab (R2019a, The MathWorks, Inc., Natick, MA, USA), run in a 4-core 16 GB RAM 3.4 GHz iMac. The command employed was ode45, which uses an explicit Runge–Kutta formula of 4–5 order (Dormand–Prince) and adaptive step size. Plots were produced in the same software and exported using an export_fig package.

## 3. Results

### 3.1. The Mathematical Model without B-Cell Development Reproduces Clinical Data

**Figure 2:**
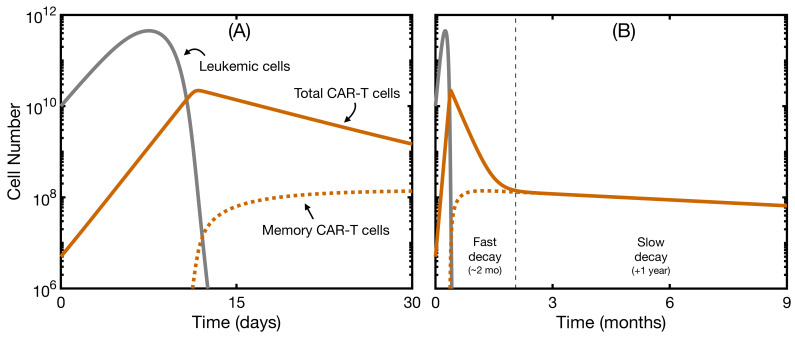
Dynamics of leukemic cells and CAR T-cells without new B-cell generation. Simulation of the evolution over time of leukemic cells (solid gray line), total CAR T-cells (solid orange line), and memory CAR T-cells (dotted orange line). In this simulation, we used only Equations (12)–(14), so we observed no recovery of B cells. The initial state was $L\left(0\right)=2\times 10^{10}$ cells, $C_{A}\left(0\right)=5\times 10^{6}$ cells, and $C_{M}\left(0\right)=0$ cells. Parameters were as in Table 1 with $\alpha =3\times 10^{-10}$ day${}^{-1}$· cell${}^{-1}$ and $h=5\times 10^{8}$ cells. (**A**) dynamics for the first month of therapy; (**B**) dynamics for the first nine months of therapy.

Most clinical data obtained in CAR T clinical trials are based on peripheral blood samples. Typical curves for CAR T dynamics can be found in refs. [22,23,24]. The usual time course of a successful therapy is as follows: After infusion, CAR T-cells are distributed around the body. Next, encounters with leukemic cells or B cells trigger activation and expansion of the CAR T population. After approximately ten days of proliferation, CAR T-cells have already expanded by two orders of magnitude or more. Following elimination of the targeT-cells, CAR T-cell numbers undergo a biphasic decline, consisting of an initial fast decay or contraction phase and a slow decay or persistence phase. Peripheral blood, however, lacks the constant production of B cells of the bone marrow, and so we expect the dynamics to be different. Therefore, in order to compare our model with data in blood, the production of B cells during the first post-therapy stages was neglected, as done in [36], and only Equations (12)–(14) were simulated. The initial state was lowered by one order of magnitude to simulate peripheral blood conditions. An example of such simulation is shown in Figure 2.

Note that, while the system is describing the bone marrow and some parameters would not appear in peripheral blood (like carrying capacity), we can still recapitulate the basics of the interaction between CAR T-cells and leukemic cells. We observed an initial phase lasting around 10 days of CAR T expansion, during which the leukemic cells still had some time to expand. After the CAR T-cells had completed a two-fold expansion, and were mainly composed of effector cells, leukemic cells decreased and eventually disappeared (Figure 2A). The CAR T-cell population then contracted rapidly as activated cells died. Memory CAR T-cells remained, accounting for the slow decay phase (Figure 2B). The magnitude of the expansion and contraction and the characteristic times involved in the response agreed with typical clinical data. This simulation allowed us to confirm the validity of the ranges chosen for the parameter values, especially the killing capacity α and the activation threshold *h*. In this way, we ensured that the results that included B-cell production were reliable.

### 3.2. Effector and Memory CAR T-Cells Are Able to Control the Disease

**Figure 3:**
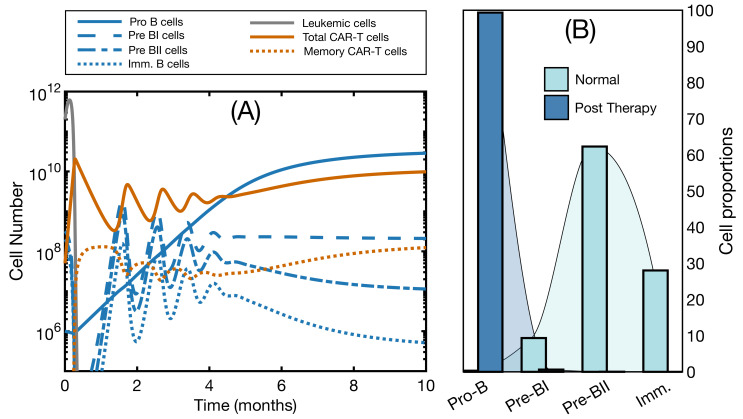
Successful control of the disease in bone marrow. (**A**) dynamics of Equations (8)–(14). Simulation now includes B cells: Pro-B cells (solid blue line), Pre-BI cells (dashed blue line), Pre-BII cells (dashed-dotted blue line), and Immature B cells (dotted blue line). Initial state is $B_{0}\left(0\right)=10^{6}$ cells, $B_{1}\left(0\right)=3\times 10^{7}$ cells, $B_{2}\left(0\right)=2\times 10^{8}$ cells, $B_{3}\left(0\right)=9\times 10^{7}$ cells, $L\left(0\right)=2\times 10^{11}$ cells, $C_{A}\left(0\right)=5\times 10^{7}$ cells and $C_{M}\left(0\right)=0$ cells. Parameters are those of Table 1 with $\alpha =3\times 10^{-10}$ day${}^{-1}$· cell${}^{-1}$ and $h=10^{9}$ cells; (**B**) proportions of B cell subsets in bone marrow, relative to the total B-cell population, under normal conditions (light blue) and after successful CAR T therapy (dark blue). Density plots are a continuous representation of the proportions, obtained with shape-preserving piecewise cubic interpolation using Matlab’s function interp1.

We next analyzed bone marrow expansion scenarios and simulated the model with the complete set of equations. Examples of the results are shown in Figure 3A. During the first 30 days, we observed behavior similar to that of Figure 2B: Early CAR expansion followed by depletion of all CD19^+^ cells, healthy and malignant. Pro-B cells increased steadily in the meantime, in response to the shortage of mature B cells. From day 30 onwards, healthy B cells started to recover. Around day 50, CAR T-cells responded to this increase: memory cells reactivated and CAR T expanded again. Cycles of CAR T expansion and B-cell reduction ensued, and, after six months, all cell types had reached a steady state and the disease had been controlled. This simulation explains the role of B cells as an endogenous vaccine. Instead of the expected number of activated cells in peripheral blood, we observed their reactivation due to CD19 antigen recovery. The steady although significantly lower level of self-renewing B cells kept CAR T-cells in a state of engagement, explaining their persistence.

An interesting prediction coming from this simulation is the change in the distribution of the B-cell population as a result of the therapy. In Figure 3B, we see the proportion of cells in each subset in homeostatic conditions and the new distribution after therapy. In the latter, B cells were mainly composed of the CD19^−^ Pro-B cell compartment, and the remaining compartments were ordered according to their maturation stage. In normal conditions, the Pre-BII compartment is the most abundant, followed by the immature compartment [38,40]. Reports of successful CAR T therapy in B-cell malignancies indicate BCA in peripheral blood as a marker of therapy response [14,15,16]. While this seems to be in contradiction with the results presented here, where we observe a recovery of B cells, this is actually due to the sensitivity of clinical detection. Indeed, here the immature compartment in the steady state after therapy is composed of approximately $10^{6}$ cells, barely 0.0002% of the total bone marrow capacity. This means that the small number of B cells in blood would go undetected. Thus, BCA in peripheral blood would be compatible with the attempts at B cell recovery in the bone marrow.

### 3.3. Initial State Does Not Affect CAR T Expansion and Outcome

**Figure 4:**
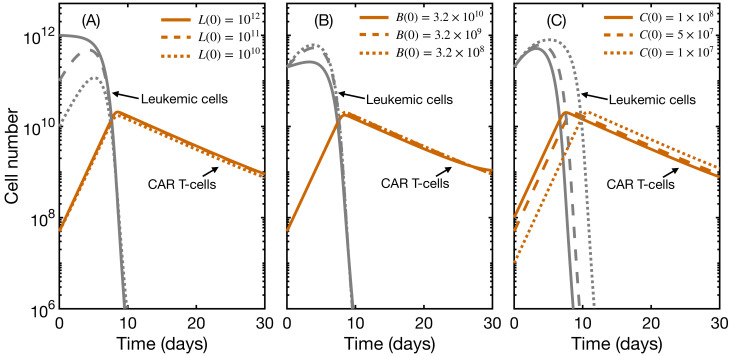
Influence of initial leukemia load, B-cell level, and CAR T dose on therapy outcome. Evolution with time of leukemic cells (gray) and total CAR T-cells (orange) for different initial configurations (In decreasing order: solid, dashed, dotted lines). When unchanged, initial values are $B_{0}\left(0\right)=10^{6}$ cells, $B_{1}\left(0\right)=3\times 10^{7}$ cells, $B_{2}\left(0\right)=2\times 10^{8}$ cells, $B_{3}\left(0\right)=9\times 10^{7}$ cells, $L\left(0\right)=2\times 10^{11}$ cells, $C_{A}\left(0\right)=5\times 10^{7}$ cells and $C_{M}\left(0\right)=0$ cells. Parameters are those of Table 1 with $\alpha =3\times 10^{-10}$ day${}^{-1}$· cell${}^{-1}$ and $h=10^{9}$ cells. (**A**) influence of leukemia burden; (**B**) influence of initial B-cell population, defined as the sum of all B-cell compartments; (**C**) influence of CAR T dose.

We next studied the effect of the initial state on therapy outcome and CAR T expansion. Clinical trials have investigated the safe dosage regime and the influence of leukemic cells and initial B-cell population. In Figure 4, we show the results obtained for different initial values for leukemic cells (A), total B cells (B), and CAR T-cells (C). None of these factors had an impact on the expansion and final outcome of the therapy, for a range of parameters justified with clinical information (see parameter estimation). The major effect was a small delay of up to three days in expansion when varying the CAR T dose by an order of magnitude. This agrees with clinical reports returning no dose–exposure relationship [14,15,16] and no clear influence of leukemia burden on CAR T expansion [22,23,24]. The explanation could lie in the fact that T-cells expand in vivo in response to stimulation, but in an antigen-independent manner [50,51], meaning that, once activated, they commit to an expansion of a given size, i.e., a constant proliferation rate This would also explain why therapy failure seems to be more closely linked to the characteristics of the product rather than to the characteristics of the disease. We will address this point in more detail in the next subsection.

### 3.4. CAR T Product Characteristics Determine Therapy Success or Failure

We have previously shown an example of disease control (Figure 3). Having noted the limited influence of the initial state on therapy outcome, we turned to CAR T product characteristics to see whether we could explain scenarios of lack of response. As discussed in the Introduction, there are two kinds of relapse, CD19${}^{-}$ and CD19^+^. In order to simulate the former, we would need to describe CD19 expression with a continuous variable or include a CD19${}^{-}$ leukemic clone compartment. We therefore restricted the analysis to CD19^+^ relapses. Clinical trials agree that non-responding patients show limited and slower CAR T expansion and shorter persistence, being unable to remove the cancer completely [14,15,16].

**Figure 5:**
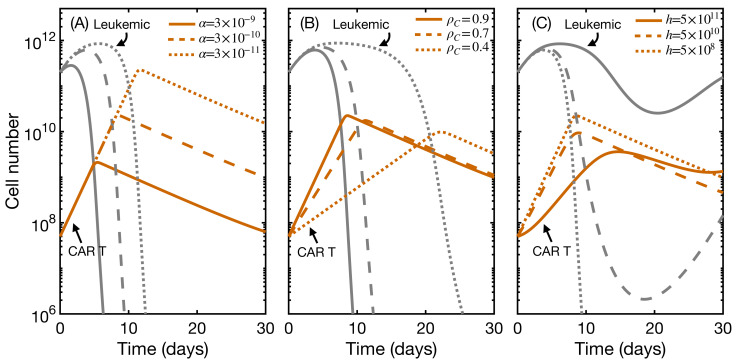
Influence of CAR T product characteristics. Evolution in time of leukemic cells (gray) and total CAR T-cells (orange) for different CAR T product attributes (In decreasing order: solid, dashed, dotted lines). Initial state is $B_{0}\left(0\right)=10^{6}$ cells, $B_{1}\left(0\right)=3\times 10^{7}$ cells, $B_{2}\left(0\right)=2\times 10^{8}$ cells, $B_{3}\left(0\right)=9\times 10^{7}$ cells, $L\left(0\right)=2\times 10^{11}$ cells, $C_{A}\left(0\right)=5\times 10^{7}$ cells and $C_{M}\left(0\right)=0$ cells. The remaining parameter values are those of Table 1 with $\alpha =3\times 10^{-10}$ day${}^{-1}$· cell${}^{-1}$ and $h=5\times 10^{8}$ cells. (**A**) influence of killing capacity; (**B**) influence of proliferation rate; (**C**) influence of activation threshold.

In order to reproduce this scenario, we explored the space of parameters delimited by α, the killing capacity of CAR T-cells; *h*, the activation threshold; and $\rho_{C}$, the proliferation rate, the three parameters related to CAR T-cells and their action. We first simulated the model for different values of one parameter while keeping the other two constant. Results for the first 30 days of treatment are shown in Figure 5. Contrary to what happened with the initial state, here we observed changes in fold expansion and time to peak expansion. Intuitively, shorter proliferation rate (B) and higher activation threshold (C) led to delayed expansion, in some cases being unable to control the malignancy. In fact, products with a high activation threshold may lead to therapy failure in cases of low presence of CD19-bearing cells (Figure S3). On the other hand, lower killing capacity implied increased expansion. The explanation is clear in the light of the model equations: when the CAR T-cells were unable to eliminate leukemic cells, the activation function F(t) remained active and proliferation continued, until there were enough CAR T-cells to reduce the leukemia burden.

**Figure 6:**
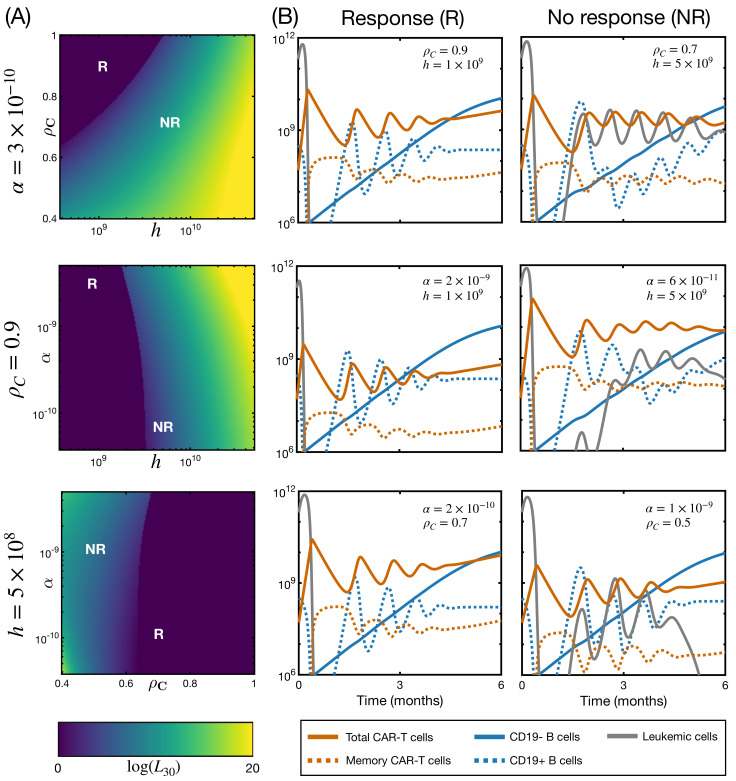
Exploration of parameter ranges for the dynamics of Responding (R) and Non–Responding (NR) patients. (**A**) number of leukemic cells at day +30, in logarithmic scale, for different regions of the parameter space. The unchanged product attribute is displayed on the *y*-axis. The remaining parameter values are those from Table 1; (**B**) examples of responding and non-responding patients for the first six months of therapy. Parameter values are marked in subfigure (**A**). Represented are activated and memory CAR T-cells (solid and dotted orange line, respectively), leukemic cells (solid gray line) and CD19${}^{-1}$ and CD19^+^ B cells (solid and dotted blue lines, respectively). The initial state of the simulations in this figure is $B_{0}\left(0\right)=10^{6}$ cells, $B_{1}\left(0\right)=3\times 10^{7}$ cells, $B_{2}\left(0\right)=2\times 10^{8}$ cells, $B_{3}\left(0\right)=9\times 10^{7}$ cells, $L\left(0\right)=2\times 10^{11}$ cells, $C_{A}\left(0\right)=5\times 10^{7}$ cells and $C_{M}\left(0\right)=0$ cells.

We next aimed to analyze the influence of simultaneous perturbation of two parameters. This can not be done as in the previous figures since it is not possible to distinguish which parameters are responsible for which changes in the dynamics. For this reason, we chose to analyze their influence not on the dynamics but on the final state of the simulation: response vs. no response. The goal was to identify regions of the parameter space related to either endpoint. In Figure 6A, we show the number of leukemic cells at day +30 ($L_{30}$), in logarithmic scale, for pairwise combinations of parameters α, $\rho_{C}$ and *h*, while keeping the excluded parameter constant. The first month is the time at which bone marrow is first monitored after infusion [14,22]. Each parameter range was discretized into 200 evenly spaced values (linearly spaced for $\rho_{C}$ and logarithmically spaced for α and *h*). We note that the killing capacity α was the least important parameter, observing more variation over the ranges of the activation threshold and proliferation rate. As seen in the previous figure, higher proliferation rates and lower activation thresholds were required for a complete response. To illustrate the evolution with the time of CAR T-cells, leukemic cells, and B cells in these scenarios, Figure 6B gives six examples of the results of the model simulations for the first six months of treatment. The three CD19^+^ B-cell compartments $B_{1}$, $B_{2}$, and $B_{3}$ are plotted together. For each heatmap, we show an example of a responding (R) and a non-responding (NR) patient. Responding patients were defined by the absence of leukemic cells at day 30 ($L_{30}<1$). Non-responding patients are defined as those with the presence of leukemic cells on that same day ($L_{30}\geq 1$). The interesting feature of this figure is in analyzing the variety of possible responses. The three responding patients displayed here showed similar behavior, comparable to that of Figure 3: Expansion followed by decay and oscillations. We found that the disease could be controlled for a range of fold expansions. The relative proportion of activated to memory cell was constant, since we were not modifying transition rates $\gamma_{AM}$ and $\gamma_{MA}$. The level of steady state CAR T-cells did change and was related to the magnitude of the initial expansion. For the non-responding patients, we observed more variety. First of all, disease recurrence can occur for different magnitudes of expansion, from one to three orders of magnitude (bottom and middle subfigures, respectively). Secondly, this recurrence is detectable from the first to the third month after infusion (top and middle subfigures, respectively). The level of B cells increased at the time of relapse, while responding patients (left column) showed no increase. This supports the reported association between BCA and therapy response (B-cell recovery indicates a possible relapse). Leukemic cells and CAR T-cells were also subject to the oscillations that were observed in responding patients, and correspond to cycles of reactivation and decay. Although this model is not reducible to a Lotka–Volterra model, due to the existence of an activation function, these cycles can be interpreted in the same way: an increase in leukemic cells occurred after the decay of activated CAR T-cells, which then regrew in response to the recurring clone. Interestingly, in some cases, the therapy was able to control the disease after relapse. This behavior has been observed in simulations of simpler models. In previous work [36], we already remarked that this scenario would not be observed in the clinical context due to the prompt actions taken after any signs of disease recurrence. Therefore, while from the dynamical point of view one could describe some cases as ‘Delayed Response’, from the clinical point of view, they would still be considered as ‘No Response.’ Finally, leukemic cells and CAR T could also coexist in equilibrium (top subfigure, NR column).

**Figure 7:**
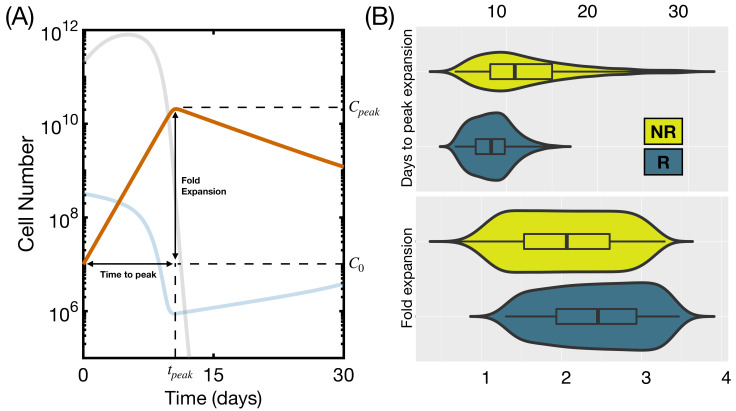
Characteristics of product expansion for Responding (R) and Non-Responding (NR) patients. (**A**) graphical representation, on a typical simulation, of the characteristics of the product expansion; (**B**) differences in time to peak and fold expansion for 4000 simulated patients with parameters from Table 1 and α in the range $5\times 10^{-11}$–$5\times 10^{-9}$ day${}^{-1}$· cell${}^{-1}$, *h* in the range $5\times 10^{9}$–$5\times 10^{11}$ cells and $\rho_{C}$ in the range 0.4–1 day${}^{-1}$. Initial state was set to $B_{0}\left(0\right)=10^{6}$ cells, $B_{1}\left(0\right)=3\times 10^{7}$ cells, $B_{2}\left(0\right)=2\times 10^{8}$ cells, $B_{3}\left(0\right)=9\times 10^{7}$ cells, $L\left(0\right)=2\times 10^{11}$ cells, $C_{A}\left(0\right)=5\times 10^{7}$ cells and $C_{M}\left(0\right)=0$ cells. The boxplot shows median and first and third quartiles.

We finally analyzed the influence of simultaneous perturbations of the three parameters. We run 4000 simulations with α, *h* and $\rho_{C}$ varying in the ranges specified throughout this section. Specifically, we took 10 evenly spaced values of $\rho_{C}$ in the interval 0.4–1; 20 logarithmically spaced values of α in the range $5\times 10^{-11}$–$5\times 10^{-9}$ and 20 logarithmically spaced values of *h* in the range $5\times 10^{8}$–$5\times 10^{10}$. We identified responding and non-responding patients as explained above and computed the level of expansion of CAR T-cells, defined as $\log(C_{peak}/C_{0})$, and the time to peak expansion $t_{peak}$ for each group. Results are shown in Figure 7. This particular range and number of simulations yielded a proportion of responding to non-responding patients of 80 to 20, similar to the early response rate recorded in clinical trials [17]. Non-responding patients took longer to achieve the maximum level of CAR cells and had lower fold expansion (Figure 7B. $p<10^{-26}$, Wilcoxon rank-sum test).

The aim of the previous two figures was to identify the parameters with the greatest influence on the outcome of the treatment. Another way to understand the relationship between them is to analyze their influence not on the outcome but on the dynamics (the values of the different variables). To perform this, we carried out Sobol’s sensitivity analysis [52] as done in a previous work [36]. This measures the fractional contribution of a single parameter to the output variance. The results show that product attributes remain the most relevant parameters (Figures S4 and S5).

### 3.5. Second Infusion in Non-Responding Patients May Improve the Therapy Outcome

A common therapeutic solution for non-responding patients is to reinfuse the original product, opening the door to the design of dose fractionation schemes. Studies of optimal combinations of timing and dosage are common in chemotherapy and radiotherapy regimes. The main difference here is that CAR T-cells expand and operate in relation to the cancer, so standard pharmacokinetic and pharmacodynamic approaches are not applicable.

**Figure 8:**
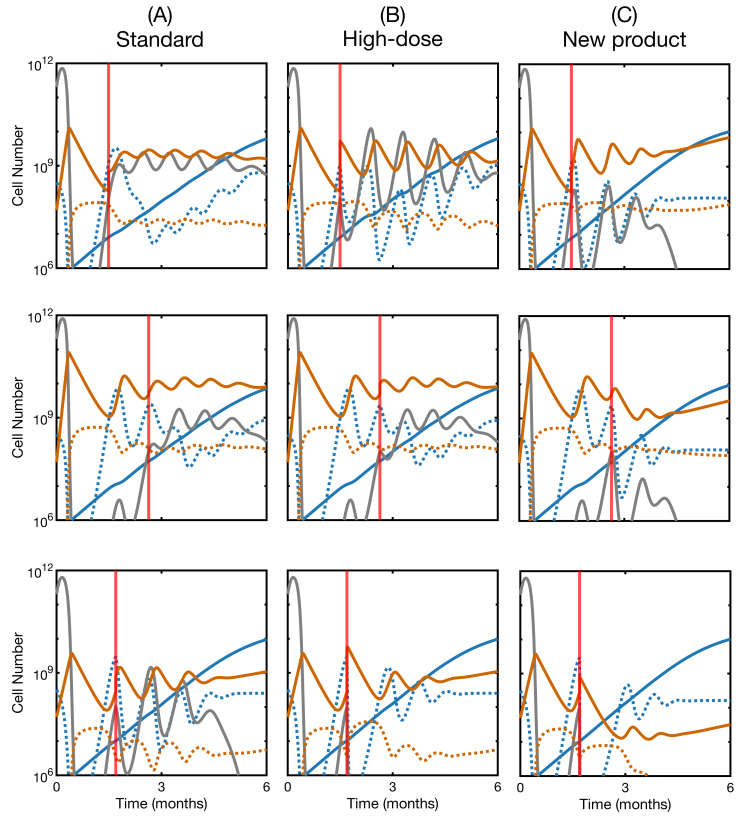
Effect of a second infusion of CAR T-cells in non-responding patients. Dynamics of leukemic cells, B cells and CAR T-cells for the three non-responding patients in Figure 6. (**A**) standard second dose of $5\times 10^{8}$ CAR T-cells; (**B**) increased second dose of $5\times 10^{9}$ CAR T-cells; (**C**) second dose of $5\times 10^{8}$ CAR T-cells of a newly-manufactured product with improved attributes. For the first patient, the new attributes were $h=5\times 10^{8}$ and $\rho_{C}=0.9$. For the second patient, the new attributes were $h=5\times 10^{8}$ and $\alpha =6\times 10^{-10}$. For the third patient, the new parameters were $\alpha =5\times 10^{-8}$ and $\rho_{C}=0.7$. We show activated and memory CAR T-cells (solid and dotted orange line, respectively), leukemic cells (solid gray line) and CD19${}^{-}$ and CD19^+^ B cells (solid and dotted blue lines, respectively). The red vertical line represents the time of the second infusion. The initial state for the simulations and the other parameter values were as in Figure 6.

We explored this issue using the mathematical model. For the three non-responding patients in Figure 6, we simulated repeated CAR T-cell therapy in three different ways. First, when the leukemia reached $10^{8}$ cells, a second dose of $5\times 10^{8}$ CAR T-cells from the original product was reinfused in silico (Figure 8A). Secondly, we did the same thing but increasing the number of cells injected in the second dose (Figure 8B). Finally, we simulated the infusion of a newly manufactured product with improved attributes (Figure 8C), according to the exploration performed in the previous subsection. We observed that a standard re-infusion was not enough to control the relapse. A higher dose could be effective in a subset of patients, while infusing an improved product turned out to be the best option. This agrees with the previous findings that the interaction of product and cancer is critical to understand the therapy outcome. Unlike chemotherapeutic drugs, which are typically modeled as a decaying concentration, this therapy expands and contracts depending on the target. These tangled dynamics are responsible for the qualitative differences with a typical dose fractionation scheme, and suggests the need for a deeper investigation of the molecular basis of T-cell and cancer cell interaction. When infusing a different improved CAR, this second product eventually became dominant in the CAR T-cell subpopulation (Figure S6). Clonal dynamics of this kind within the product have already been reported [53], opening the door to an evolutionary exploration of the therapy, another feature that is absent in other types of treatment.

To explore the full range of product attributes, we repeated the simulations shown in Figure 6 with the reinfusion protocol. As explained above, a second dose was given when leukemia reached $10^{8}$ cells or the highest value below that number. Where there was no initial response, a second dose was infused computationally at day +30. The results showed that the regions associated with success and failure remained invariant, reinforcing the conclusion that a single reinfusion of the original product would not be a successful strategy in general (Figure S7).

## 4. Discussion

CAR T-cell therapy is the most promising therapeutic option for recurrent B-cell acute lymphoblastic leukemia in children. The recent approval of the first drug by the US and EU drug administrations has culminated three decades of research in adoptive transfer of autologous, genetically modified T-cells. Clinical trials have shown good rates of response and long-term remission. After four generations, the CAR T construct has achieved enough expansion and persistence potential to induce successful responses. The persistence of CAR T-cells in the organism, acting as a surveillance mechanism, is thought to be promoted by the continuous generation of B cells in bone marrow, acting as an endogenous vaccine [24]. The interaction between renewing B cells, CAR T-cells, and leukemic cells in the bone marrow is therefore likely to predict the outcome of the therapy. The lack of extensive bone marrow data due to the more invasive way it must be extracted means that other means of studying these dynamics must be explored.

In this study, we aimed to incorporate into mathematical models two elements that have been reported to be important by clinical research. The first was the role of bone marrow as a source of CAR T-cell ongoing stimulation, due to the production of B cells. The second was the kinetics of the product, which had been characterized by a biphasic decline and explained by the existence of two CAR T subpopulations, one effector, the other memory. This was done by building on previous mathematical models of B cell development and CAR T therapy, which had underlined the importance of B cell production in determining therapy outcome. Contrary to the usual mass action term for the proliferation of the CAR T-cells, we followed previous models of T-cell response and included an activation function. In this way, we accounted for the antigen-independent features of T-cell response. With this, we mean that T-cells commit to a given clonal expansion upon activation; the role of the antigen is the initiation of this process and not the determination of the proliferation rate, as would happen in Lotka–Volterra models. Antigen thus functions as an on-off switch (we show this in Figure S8 for simulations in Figure 2 and Figure 3). The result of these assumptions was a mathematical model of seven ordinary differential equations, with many of the parameters already studied or available from clinical data.

Our first goal was to reproduce the known kinetics of the product and the clinical observations related to the therapy. By omitting the equations related to B-cell production, we were able to recover the characteristic curves reported from clinical trials with the biphasic decline in the number of CAR T-cells. We then simulated the production of B cells, describing the differences with the dynamics in peripheral blood. We observed the reactivation of CAR T-cells due to the recovery of B cells and the achievement of a steady state which accounted for both the persistence of the drug and the absence of B cells in blood. The model predicted relative proportions of B-cell subsets and CAR T subsets directly attainable from the flow cytometry data of long-term remission patients. In particular, the reorganization of the B-cell subsets, with a predominant CD19${}^{-}$ compartment, has recently been reported in patients who respond to anti-CD19 therapy [54]. This otherwise unnoticeable precursor could reach up to 100% of all the B-cell precursors, as shown here. We also noted that peripheral blood and bone marrow dynamics were similar during the first month of therapy, clarifying the relevance of this time scale in assessing response. One of the first clinical trials described recovery of hematogones or normal B-cell progenitors in bone marrow from day 28 onwards, which matches the simulations of our model [15].

Our second goal was to identify which parameters were most relevant in relapse. We first assessed the importance of the initial quantities of B cells, leukemic cells, and CAR T-cells. None of these factors seemed to influence expansion and outcome. With respect to leukemia burden or initial B-cell population, clinical trials have reported either no influence or contradictory evidence [22,24,55,56]. There is more agreement about the lack of relationship between dose and response [57]. We then evaluated the influence of CAR T product characteristics, with the hypothesis that some phenotypic properties of the CAR T-cells are associated with lack of response [58]. We showed that proliferation rate $\rho_{C}$ and activation threshold *h* are relevant in determining response. This seems reasonable, since these are the two factors that have been the subject of improvement in the different generations of CAR T-cells. In fact, early generations failed due to impaired activation and expansion [59]. We also showed that a high activation threshold could explain those cases in which low antigen burden is associated with therapy failure [58]. On the other hand, we observed rather counterintuitive behavior when varying the killing capacity α. Lower values of killing capacity meant increased expansion, due to the continuous activation caused by leukemic cells that the drug was not able to eliminate. This contradicts the observed fact that non-responding patients have reduced expansion. Nonetheless, when the whole range of parameters was evaluated, we found significant differences in expansion between the groups of responding and non-responding patients. In addition, in some cases, non-responding patients expand normally. CD19${}^{+}$ relapses could then be explained by the impaired effector capacity of the CAR T-cells. The literature suggests that this could be induced by leukemic cells themselves [60], which is something to explore in future works. Non-responding patients were also shown here to reach maximum expansion later than responding patients, which also agrees with clinical observations. In this case, however, clinical differences were more pronounced [6]. In support of this counterinituitive result, some studies have shown that a product with lower affinity, which here means a lower killing capacity, can yield better results in expansion and persistence [61,62]. Finally, the sensitivity analysis showed that parameter α was especially important for the dynamics of CAR T-cells, while *h* and $\rho_{C}$ were more relevant for the dynamics of B-cells and leukemic cells. Note, however, that a parameter can have influence on the dynamics but not on the endpoint of the therapy, as happened with parameter α.

We then used the mathematical model to test, in silico, the effectivity of a second dose of CAR T, a procedure that has been carried out in patients that did not respond to the first infusion. We showed that the same product is unlikely to control the disease, while increasing the dose, and especially improving the quality of the product, could be more impactful. Clinical trials have precisely reported little to no success of reinfusions [15,16,63], which could also be related to the lack of lymphodepleting chemotherapy and/or the role of regulatory T-cells. A recent study identified the factors associated with complete response after second infusions [64]. These are mainly a higher dose, as reported here, and lymphodepleting chemotherapy before the first dose. Failure of the second dose has also been attributed to the development of an immune response against the CAR T [65]. We showed here that it could be due to the dynamical nature of the interaction: The outcome is determined by the parameters that control this interaction, and not by the amounts involved or by their timing, as would happen with chemotherapy. For this reason, rather than dose-fractioning schemes, the optimal course of action would be to search for ways to affect this interaction. For example, infusions of T-cell specific antigen-presenting cells have been shown to boost anti-tumor response [58,66]. In this line, the model predicts that infusion of B-cells upon relapse could potentially control the disease or delay recurrence in some cases (Figure S9).

A recent review of mathematical models of CAR T-cell therapy [25] suggested a number of requisites that any of these models should include to provide a faithful description of the biological dynamics. The model presented here succeeds in recapitulating antigen-driven expansion, bi-exponential decay, and a limited expansion despite large antigen burden. It does so without depending on a piecewise definition of the model, integrating both expansion and decay in the same mathematical framework. The model also allowed for the accommodation of multiple dosages. A point of disagreement with this review was the oscillatory behavior of CAR T-cells and B cells, declared as an undesirable feature. In our case, they can be explained analogously to predator–prey dynamics, although the system is not directly reducible to a Lotka–Volterra system of equations. Interestingly, behavior of this kind in bone marrow has been recently reported by a clinical trial [67]. In addition, these cycles were predicted to occur in the bone marrow, where B cells are produced, and not in peripheral blood, which is where the kinetics of the product are normally assessed. Given the length and amplitude of the cycles shown here, current protocols of bone-marrow extraction might not be able to detect this phenomenon within the precision of cell abundance determinations.

The model presented here, despite its success in representing a range of clinical observations, is missing some features of non-responding patients. First of all, CD19${}^{-}$ relapses demand a different modeling approach, or the addition of more compartments, possibly including Darwinian selection processes like preexisting CD19${}^{-}$ leukemic clone selection or CD19 downregulation [68]. With respect to CD19^+^ relapses, the model was not able to capture long-term relapse, occurring from month three onwards. We have observed here that, from this date, the system typically reaches a steady state, in coexistence with either healthy B cells (in responding patients) or both leukemic clone and B cells (in non-responding patients). A steady state of this kind is consistent with patients in whom BCA has been ongoing for long periods of time, but not those who either relapse or recover B cells in peripheral blood in the meantime. In addition, differences in expansion and time to maximum expansion between responding and non-responding patients should be larger. This may be due to our arbitrary selection of parameter values in their respective ranges, or to the fact that we did not take into account correlations between them. Nonetheless, we cannot discard the possibility that other processes are at work.

Some biological elements may be needed in more exhaustive mathematical models, able to account for more biological phenomena present in CAR T-cell treatments. First, mechanisms of T-cell exhaustion could help in accounting for long-term relapses. These mechanisms are thought to be linked to excessive stimulation or activation [69], and are often found in persistent infections such as lymphocytic choriomeningitis (LCM) or cytomegalovirus (CMV) [70]. A mechanism of this kind would attenuate the excessive expansion in patients with lower killing capacity, leading to the death of CAR T-cells subjected to continuous stimulation. It should also explain the loss of the CAR T-cells in situations of coexistence with the leukemic cell population, which demands sustained activation and effector functions. Finally, it should also account for the loss of the product after longer periods of coexistence with B cells, which are not as demanding to the CAR T as the clone from the immunological point of view. This would also cause the attenuation of the cycles of expansion and decay.

A second biological element that should be accounted for in mathematical models is a more detailed description of the process of activation, differentiation, and renewal of CAR T-cells. This part of the system has been shown to be more significant than, for example, the compartmentalization of B-cell development or the initial state. Along these lines, there are studies that explain how the specific subsets of T-cells in the initial leukapheresis can be a determinant of the response. For example, central memory T-cells can yield a more effective product than other phenotypes [17]. This suggests that, while a separation in effector and memory T-cells is enough to represent the kinetics of the drug, a more detailed description of the subsets could be necessary to explain and interpret therapy outcome. One last aspect of interest is the influence of CD19 surface density on activation and elimination. While this has not been reported to have a significant influence [71], contrary to other markers like CD22 [72], infused product data could help elucidate its role. This feature could be included in a more complex model that incorporates CD19 expression as another variable.

## 5. Conclusions

We have presented a mathematical model of CAR T-cell therapy in bone marrow that is able to explain a number of clinical features that have been observed. It can account for the measured kinetics of the drug and for the coincidence of BCA and CAR T persistence. It also agrees in attributing more relevance to product characteristics in determining response. Some of the predictions and properties of the model are easily testable from already existing clinical data, for example by adding panels for B cells and T-cells in flow cytometry data. Relative proportions of these cellular subpopulations could help in parameterizing this and other models of CAR T therapy response. The mathematical model also suggests the importance of characterizing the composition of both the infused product and the patient’s initial T-cell repertoire. Simple improvements of the mathematical model could explain additional observations, possibly including a more detailed description of the T-cell compartment and the consideration of mechanisms of T-cell exhaustion.

We hope that this study will stimulate mathematical research in this interesting area, in which in-silico modeling can help in finding optimal therapeutic schedules and in suggesting ways for improving treatments with these promising immunotherapies.

## Figures and Tables

**Table 1:** Parameter values for the biomathematical model given by Equations (8)–(14).

Parameter	Meaning	Value	Units
$\rho_{0}$	Pro-B proliferation rate	ln(2)/8	day${}^{-1}$
$\rho_{1}$	Pre-BI proliferation rate	ln(2)/1	day${}^{-1}$
$\rho_{2}$	Pre-BII proliferation rate	ln(2)/1.5	day${}^{-1}$
$\gamma_{0}$	Transition rate: Pro-B to Pre-BI	0.02	day${}^{-1}$
$\gamma_{1}$	Transition rate: Pre-BI to Pre-BII	0.168	day${}^{-1}$
$\gamma_{2}$	Transition rate: Pre-BII to Immature	0.144	day${}^{-1}$
$\gamma_{3}$	Blood exit rate	0.288	dayt${}^{-1}$
*k*	Signal intensity	$10^{-10}$	cell${}^{-1}$
$\rho_{L}$	Leukemic cell proliferation rate	$\rho_{1}$	day${}^{-1}$
$L_{max}$	Leukemic cell carrying capacity	$10^{12}$	cell
$\gamma_{L}$	Leukemic cell blood exit rate	$0.001\cdot \gamma_{3}$	day${}^{-1}$
α	Activated CAR T killing capacity	$3\times 10^{-9}$–$3\times 10^{-11}$	day${}^{-1}$· cell${}^{-1}$
$\rho_{C}$	Activated CAR T proliferation rate	0.9	day${}^{-1}$
$\tau_{A}$	Activated CAR T mean lifetime	6.5	day
$\gamma_{AM}$	Activated to memory transition rate	0.001	day${}^{-1}$
$\gamma_{MA}$	Memory to activated transition rate	0.33	day${}^{-1}$
$\tau_{M}$	Memory CAR T mean lifetime	300	day
*h*	CAR T activation threshold	$10^{8}$–$10^{11}$	cell

## Data Availability

Not applicable.

## Supplementary Materials

The following are available at https://www.mdpi.com/article/10.3390/ijms22126371/s1.

## Abbreviations

The following abbreviations are used in this manuscript:

CAR	Chimeric Antigen Receptor
ALL	Acute Lymphoblastic Leukemia
CD	Cluster of Differentiation
BCA	B-Cell Aplasia
FDA	Federal Drug Administration
