# Supplementary material for: A Mathematical Description of the Bone Marrow Dynamics during CAR T-Cell Therapy in B-Cell Childhood Acute Lymphoblastic Leukemia
Source: Int J Mol Sci. 2021 Jun 14;22(12):6371. doi: 10.3390/ijms22126371 (PMC8232108; doi:10.3390/ijms22126371)
Supplement: Supplementary file 1 [file ijms-22-06371-s001.zip › ijms-1237572-Final-Supplement-Material.pdf]

A Mathematical Description of Bone Marrow Dynamics during CAR-T Cell Therapy in B-cell Childhood Acute Lymphoblastic Leukemia

Supplementary Information

Álvaro Martínez-Rubio [1,2]*, Salvador Chulián [1,2], Cristina Blázquez Goñi [2,3], Manuel Ramírez Orellana [4], Antonio Pérez Martínez [5,6], Alfonso Navarro-Zapata [5], Cristina Ferreras [5], Victor M. Pérez-García [7] and María Rosa [1,2]

[1] Department of Mathematics, Universidad de Cádiz, Puerto Real, Cádiz, Spain
[2] Biomedical Research and Innovation Institute of Cádiz (INiBICA), Hospital Universitario Puerta del Mar, Cádiz, Spain
[3] Department of Pediatric Hematology and Oncology, Hospital de Jerez Cádiz, Spain
[4] Department of Paediatric Haematology and Oncology, Hospital Infantil Universitario Niño Jesús, Instituto Investigación Sanitaria La Princesa, Madrid, Spain
[5] Translational Research in Pediatric Oncology, Hematopoietic Transplantation and Cell Therapy, IdiPAZ, Hospital Universitario La Paz, Madrid, Spain
[6] Pediatric Hemato-Oncology Department, Hospital Universitario La Paz, Madrid, Spain
[7] Department of Mathematics, Mathematical Oncology Laboratory (MOLAB), Universidad de Castilla-La Mancha, Ciudad Real, Spain

∗ alvaro.martinezrubio@uca.es

1 Parameter estimation

Due to the difficulty in obtaining direct estimates for each parameter in the model, we focused on obtaining plausible orders of magnitude, ensuring the correct relationship between parameter values (e.g. which proliferation rate is higher for B-cell subsets) and comparing the resulting dynamics with known dynamical characteristics such as reconstitution times after autologous bone marrow transplantation.

For the case of the B-cell subsets, we previously studied Eqs (2b)-(2d) in [1]. Briefly, in vitro studies show that proliferation rates are of the order of magnitude of days [2, 3, 4, 5]. Upon CD19 acquisition there is sustained proliferation that decreases as the cell matures [5, 6, 7], which justifies the choice $\rho_1 < \rho_2$. With respect to the B_0 compartment, we chose a slower proliferation rate, in accordance with the idea that more immature hematopoietic progenitors cycle at slower rates [8, 9]. Exact values were set after simulating and comparing with the fact that B-cell progenitors can be detected in bone marrow at 1 month after transplantation [10, 11]. Finally, transition rates can be derived from studying steady state conditions as explained in [1], and the values are selected to match known steady state proportions (These can be consulted in [12, 13]), assuming a homeostatic B-cell amount of the order of magnitude of 10^{10} cells (see [1] for the derivation). Figure S1 (A,B) shows a simulation of Eqs (2a)-(2d) with the parameters specified in Table 1, reflecting the biological evidence referenced above (reconstitution times and relative proportions). With respect to the leukemic cell population, for simplicity we assumed it inherits the proliferation rate of the cell of origin, being its exaggerated growth due to lack of self-inhibition. The exit rate is set lower than normal B-cell exit rate so that they do not accumulate in blood at exaggerated levels. Finally, assuming a fully invaded bone marrow can exceed its normal homeostatic capacity, we set the saturation value at approximately twice this amount (see [14] for reference values). Figure S1 (C,D) shows a simulation of the growth of the leukemia and its rapid increase in the absence of treatment.

Finally, most of the parameters of the CAR T-cell population can be obtained from experimental fitting of clinical data [15]. These include proliferation rate, effector to memory transition rate and death times for both phenotypes. For the memory to activated transition rate we assumed a characteristic time of 3 days, given the high intensity of secondary responses [16]. This parameter only controls the final level of memory CAR T-cells and thus would not influence the dynamics. Lastly, parameters α and h lack a direct experimental measure. In order to obtain plausible ranges, we simulated peripheral blood conditions as explained in Section 3.1 for different orders of magnitude. Figure S2 shows some of the scenarios that were discarded due to unrealistic biological conditions. More precisely, for $\alpha = 3 \cdot 10^{-8}$ the product drops too much below the initial value, and for $\alpha = 3 \cdot 10^{-12}$ it expands to unrealistic levels. Likewise, for $h = 5 \cdot 10^{13}$ the product drops unrealistically, and for $h < 5 \cdot 10^{7}$ there are no significant differences since CAR T-cells become activated just as quickly.

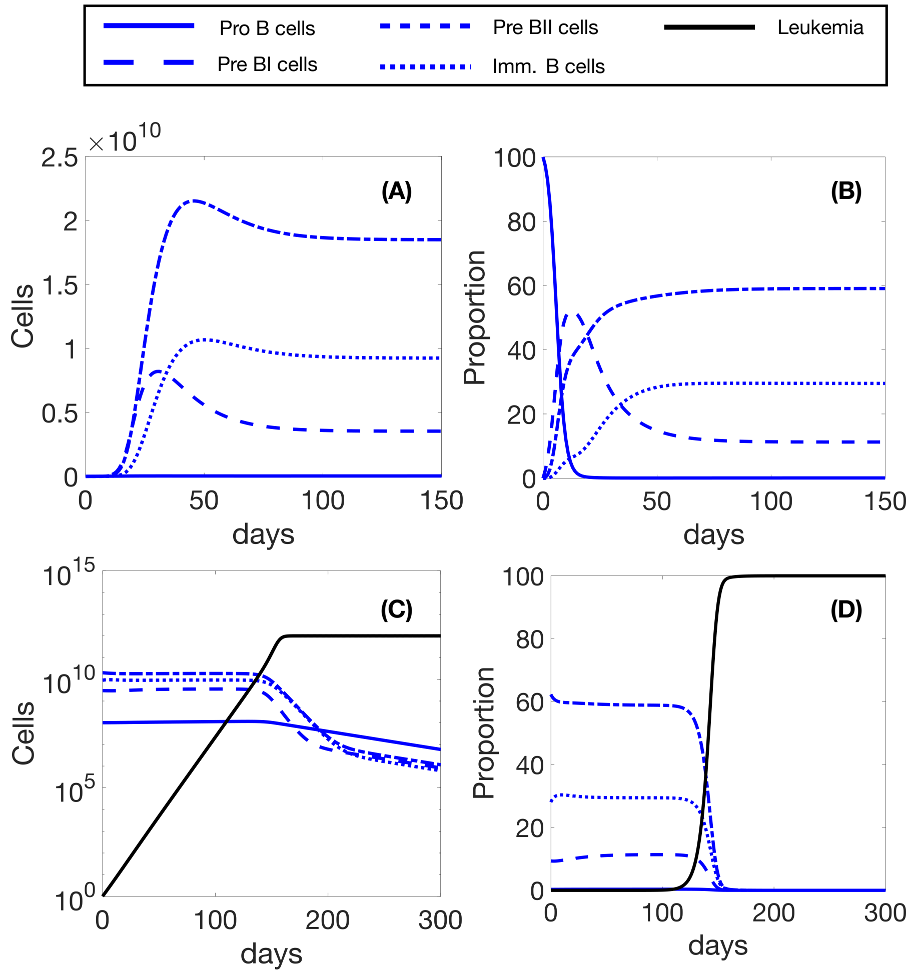

Figure S1: Simulations of hematopoietic and leukemic compartments. **(A,B)** Evolution in time and relative proportions of Pro-B, Pre-BI, Pre-BII and immature B-cells simulating immune reconstitution after transplant. Parameters from Table 1. Initial state $B_0(0) = 10^7$, $B_1(0) = 0$, $B_2(0) = 0$, $B_3(0) = 0$. **(C,D)** Evolution in time and relative proportions of B-cell compartments and leukemic cells. Parameters from Table 1. Initial state $B_0(0) = 10^8$, $B_1(0) = 3 \cdot 10^9$, $B_2(0) = 2 \cdot 10^{10}$, $B_3(0) = 9 \cdot 10^9$, $L(0) = 1$.

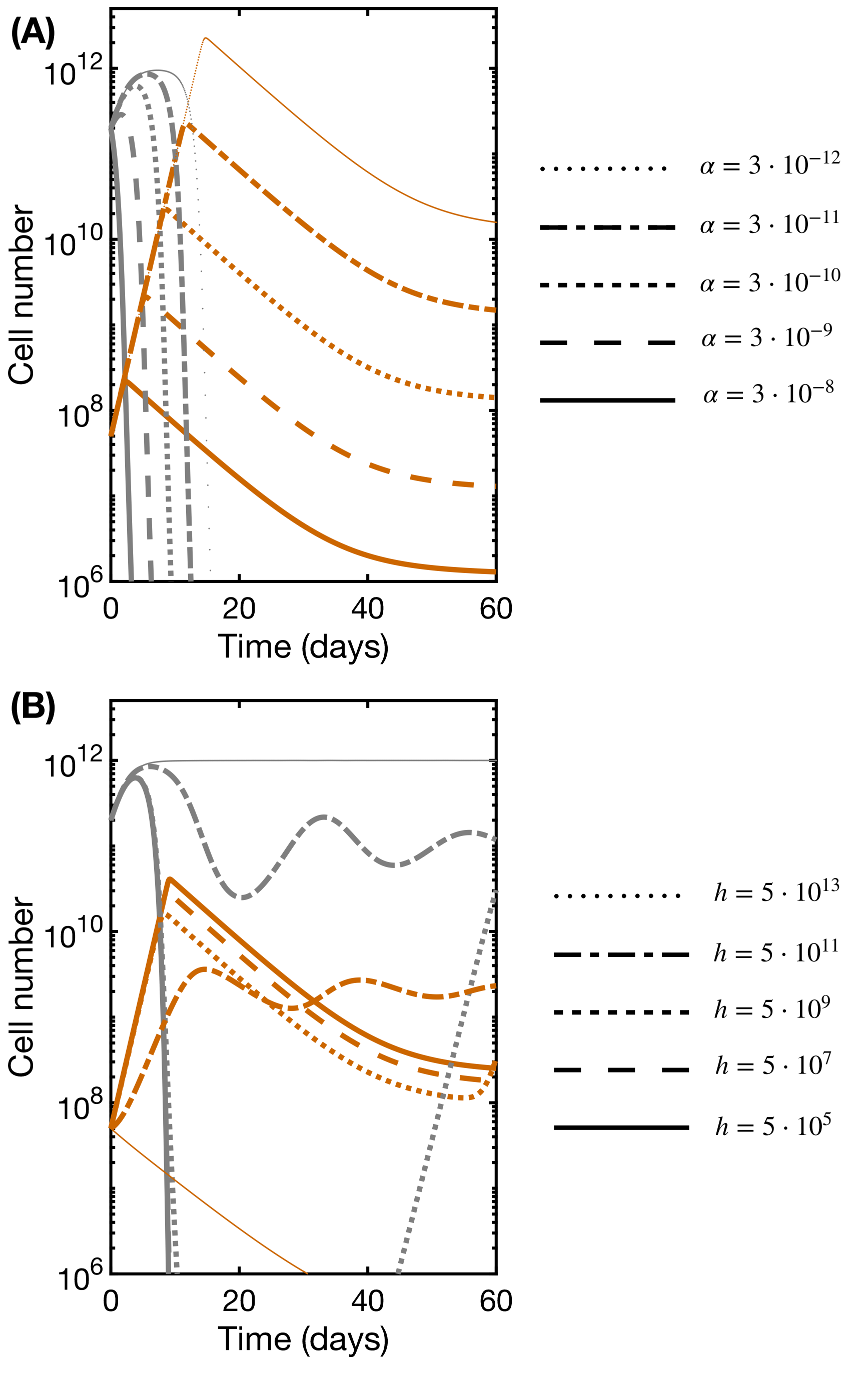

Figure S2: Parameter ranges for α and h. Simulations of the scenario described in Figure 2 for different ranges of parameters **(A)** α and **(B)** h. The rest of parameters and initial state are as in Figure 2.

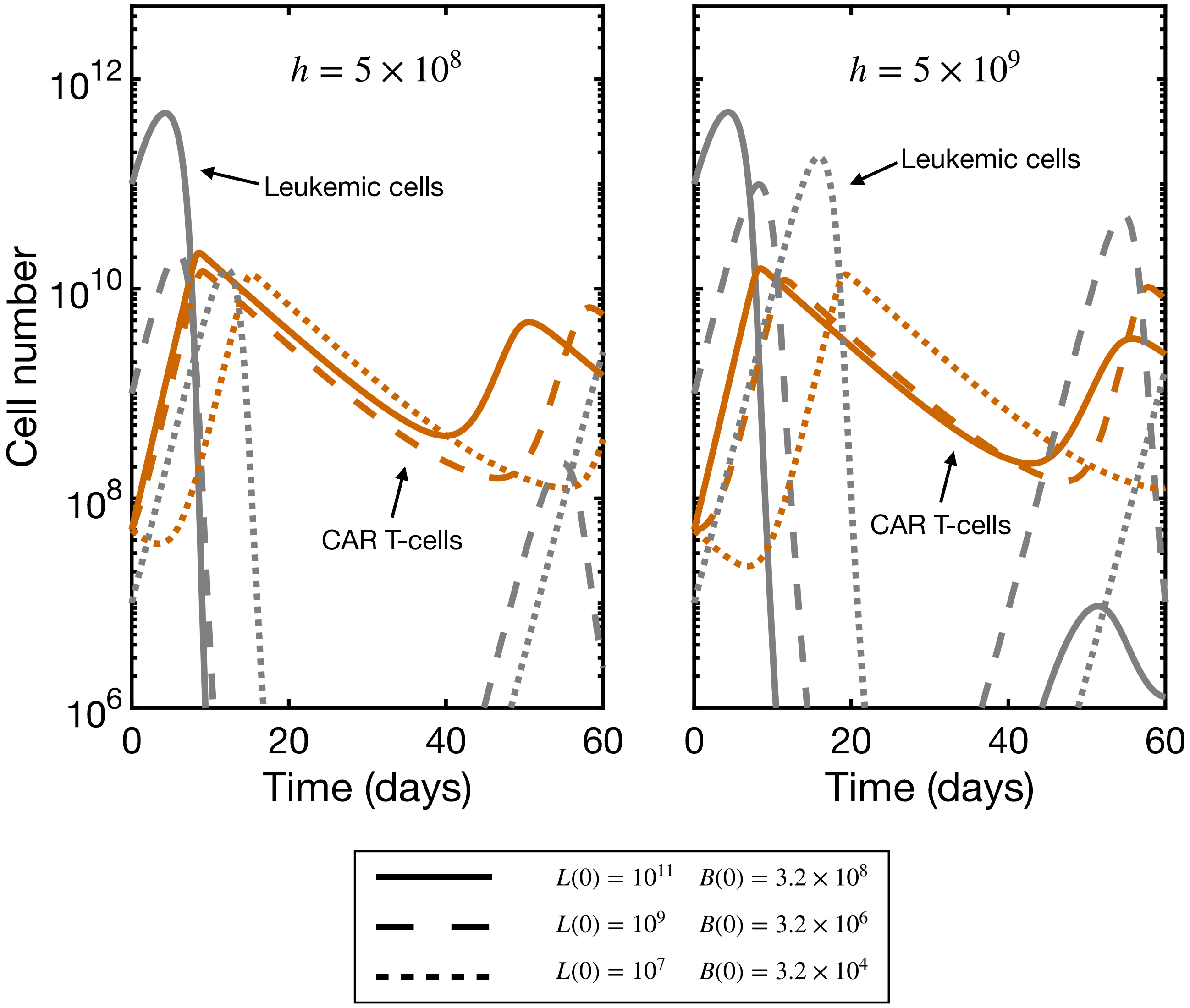

Figure S3: Influence of low CD19 burden. Evolution in time of leukemic cells (gray) and total CAR T-cells (orange) for different initial configurations (In decreasing order: solid, dashed, dotted lines). Parameters are those of Table 1 with $\alpha = 3 \times 10^{-9}$ day^{-1} $\cdot$ cell^{-1}. In general, leukemia recurrence is more frequent and occurs sooner for higher values of activation threshold and lower CD19 burden.

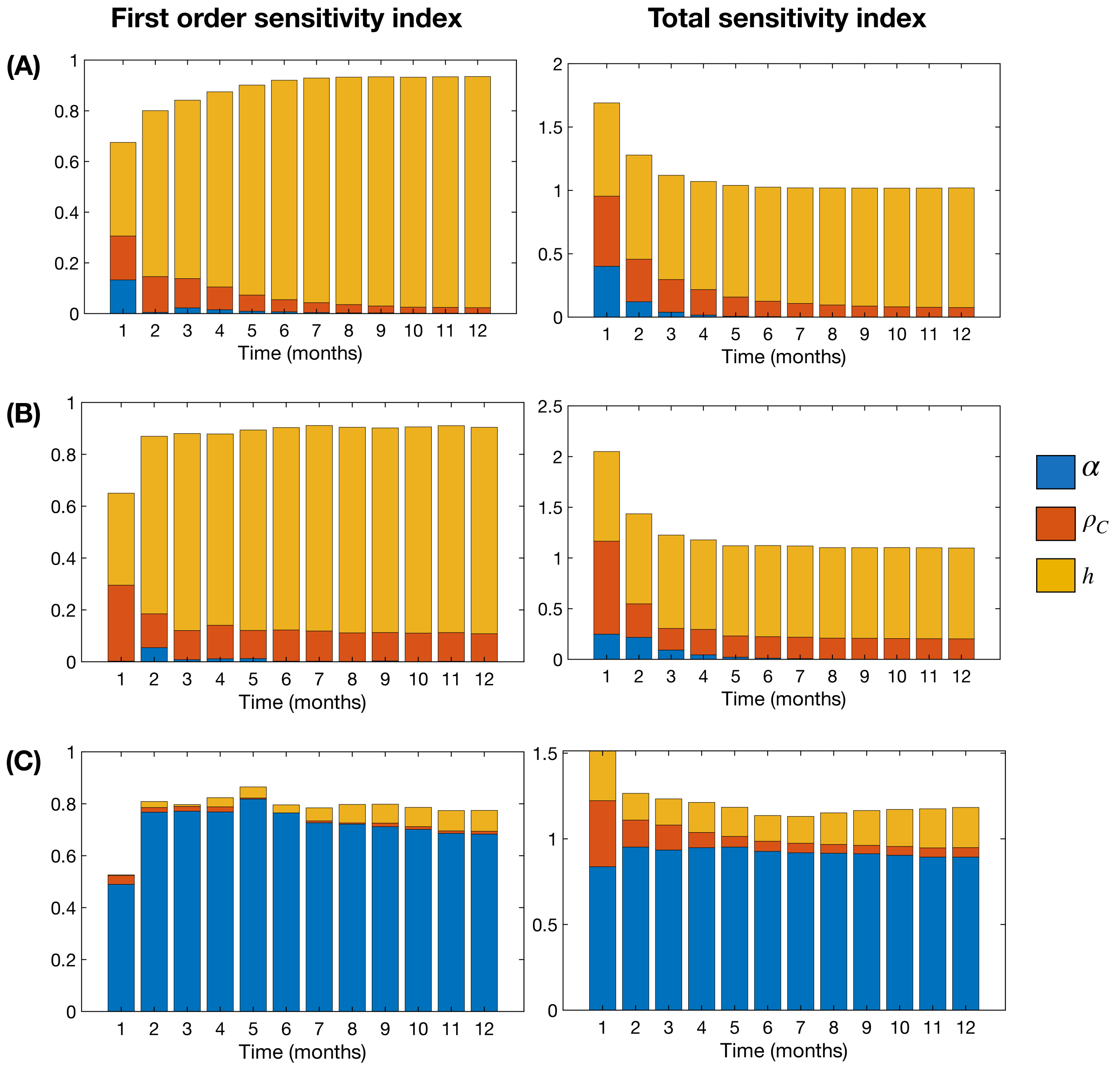

Figure S4: Sobol's sensitivity analysis for product attributes. Fractional contribution of CAR T-cell product attributes α, ρ_C and h on the model's output variance for one year of treatment. 1500 simulations were performed with each parameter taking random values from the following intervals: α in $[5 \cdot 10^{-11}, 5 \cdot 10^{-9}]$, ρ_C in $[0.4, 1]$ and h in $[5 \cdot 10^{9}, 5 \cdot 10^{11}]$. The rest of parameters values are those of Table 1. **(A)** Influence on total B-cell population. **(B)** Influence on leukemic cell population. **(C)** Influence on total CAR T-cell population.

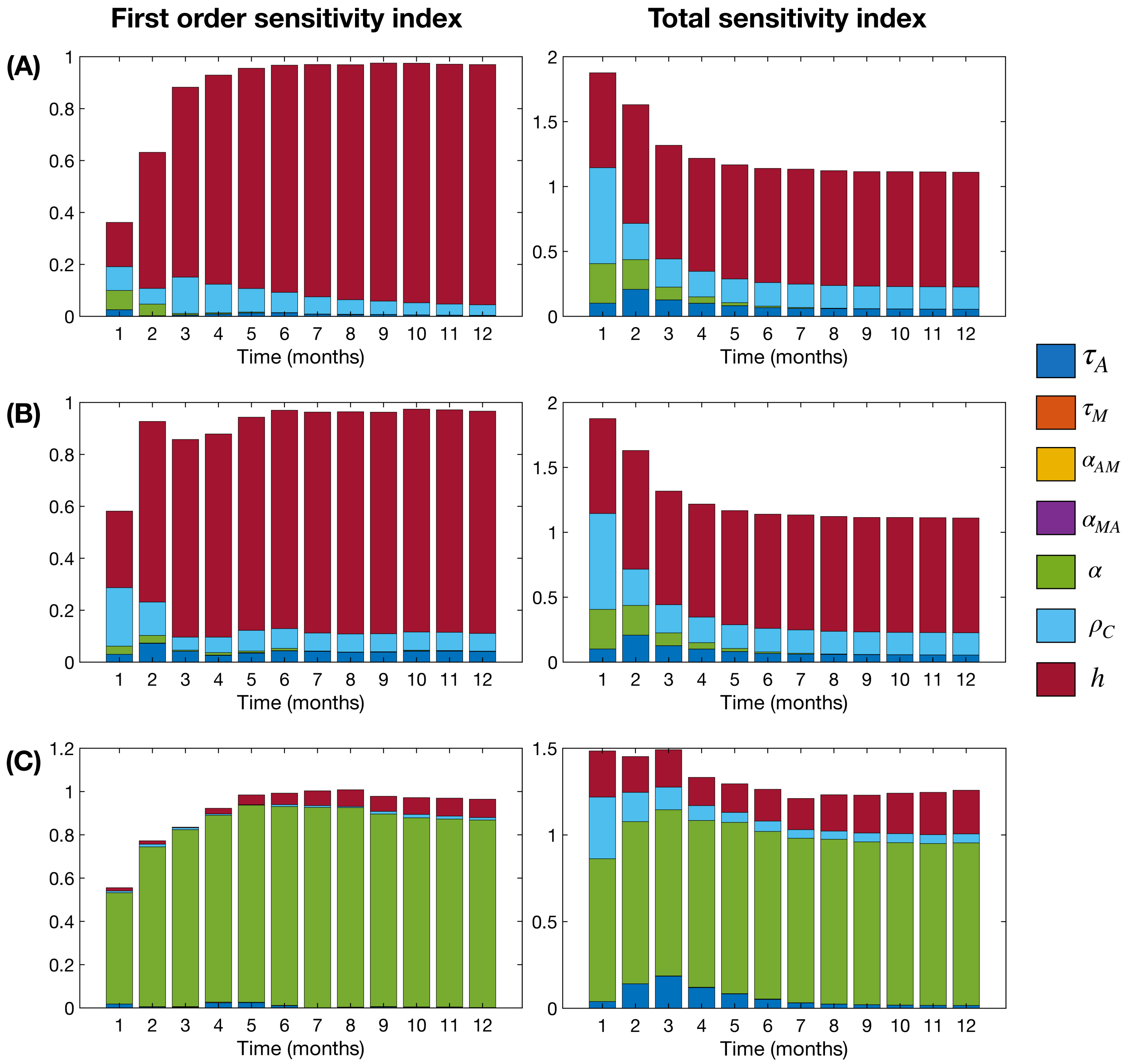

Figure S5: Sobol's sensitivity analysis for therapy parameters. Fractional contribution of CAR T-cell therapy parameters on the model's output variance for one year of treatment. 1500 simulations were performed with each parameter taking random values from the following intervals: τ_A in $[5, 8]$, τ_M in $[150, 600]$, α_{AM} in $[0.0005, 0.0015]$, α_{MA} in $[0.15, 1]$, α in $[5 \cdot 10^{-11}, 5 \cdot 10^{-9}]$, ρ_C in $[0.4, 1]$ and h in $[5 \cdot 10^{9}, 5 \cdot 10^{11}]$. The rest of parameters values are those of Table 1. **(A)** Influence on total B-cell population. **(B)** Influence on leukemic cell population. **(C)** Influence on total CAR T-cell population.

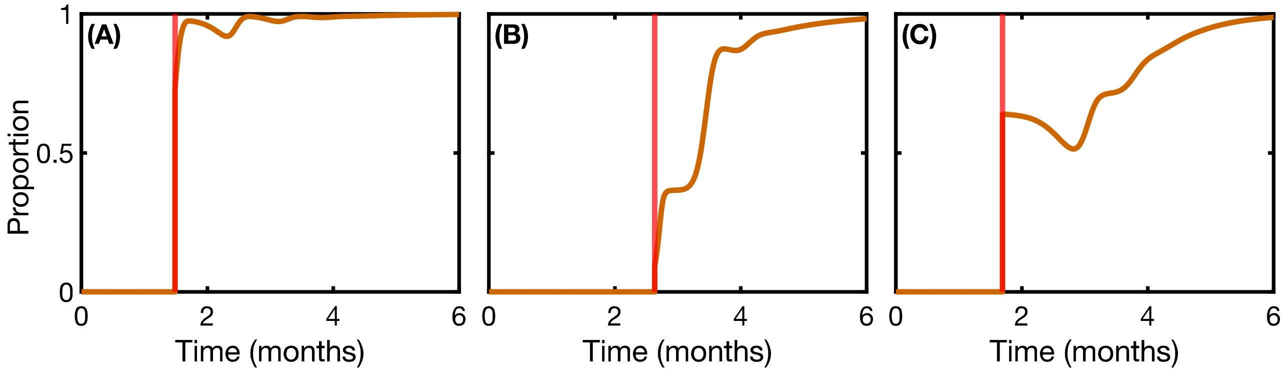

Figure S6: Proportion of newly manufactured CAR after second infusion. Evolution of the proportion of the second CAR infused after lack of response to first infusion, for the case of the improved product. The three cases correspond to the three examples shown in Figure 7, with new attributes **(A)** $h = 5 \times 10^8$ and $\rho_C = 0.9$, **(B)** $h = 5 \times 10^8$ and $\alpha = 6 \times 10^{-10}$, **(C)** $\alpha = 5 \times 10^{-8}$ and $\rho_C = 0.7$. Rest of parameters as in Figure 7. Simulations were done by performing two runs of the model, changing the parameters relative to the product in the second one and using the final state of the first run as initial state of the second.

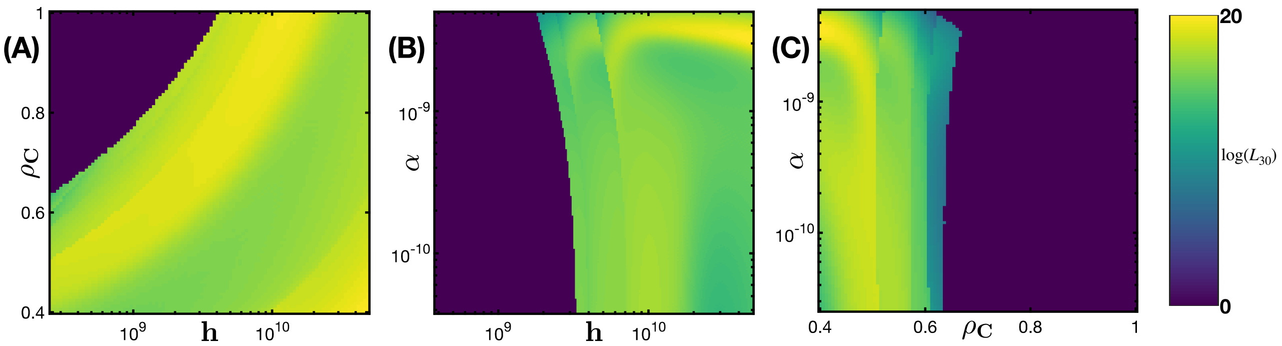

Figure S7: Exploration of parameter ranges for response to second infusion. Number of leukemic cells at day +30 after the second infusion of 5×10^8 CAR T-cells of the initial product, in logarithmic scale. Unchanged parameters and initial state as in Figure 6. Discontinuities correspond to the thresholds (in either cell number or time) set for the moment of reinfusion. Subfigures (A), (B) and (C) correspond to the three heatmaps in Figure 6: Top, middle and bottom respectively.

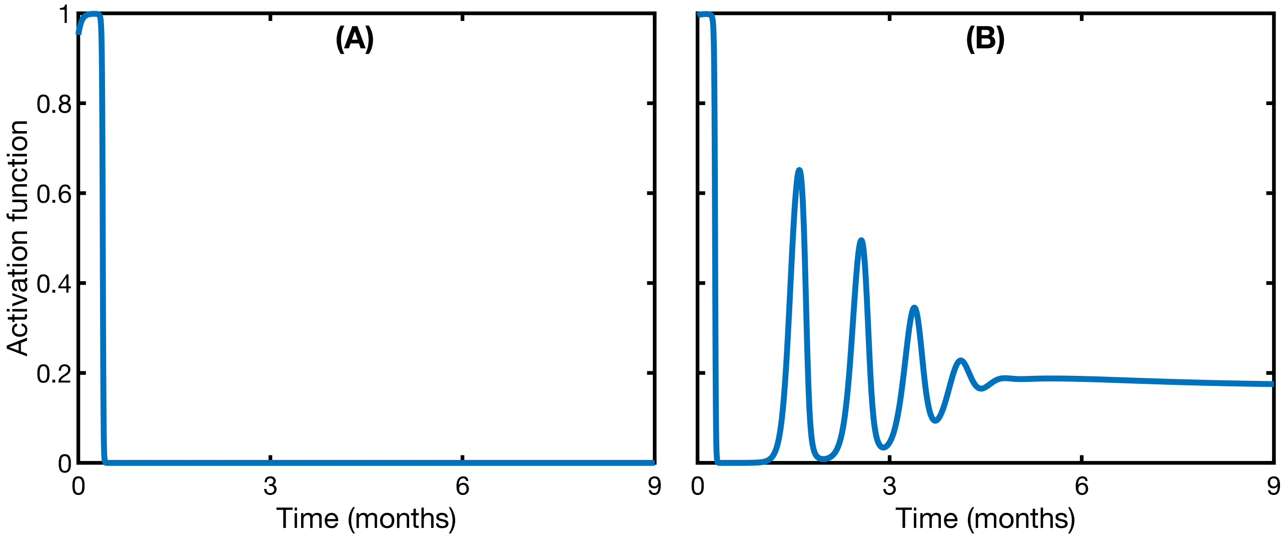

Figure S8: Evolution in time of activation function. (A) Activation function for peripheral blood simulation (Figure 2 in main text). **(B)** Activation function for bone marrow simulation (Figure 3 in main text).

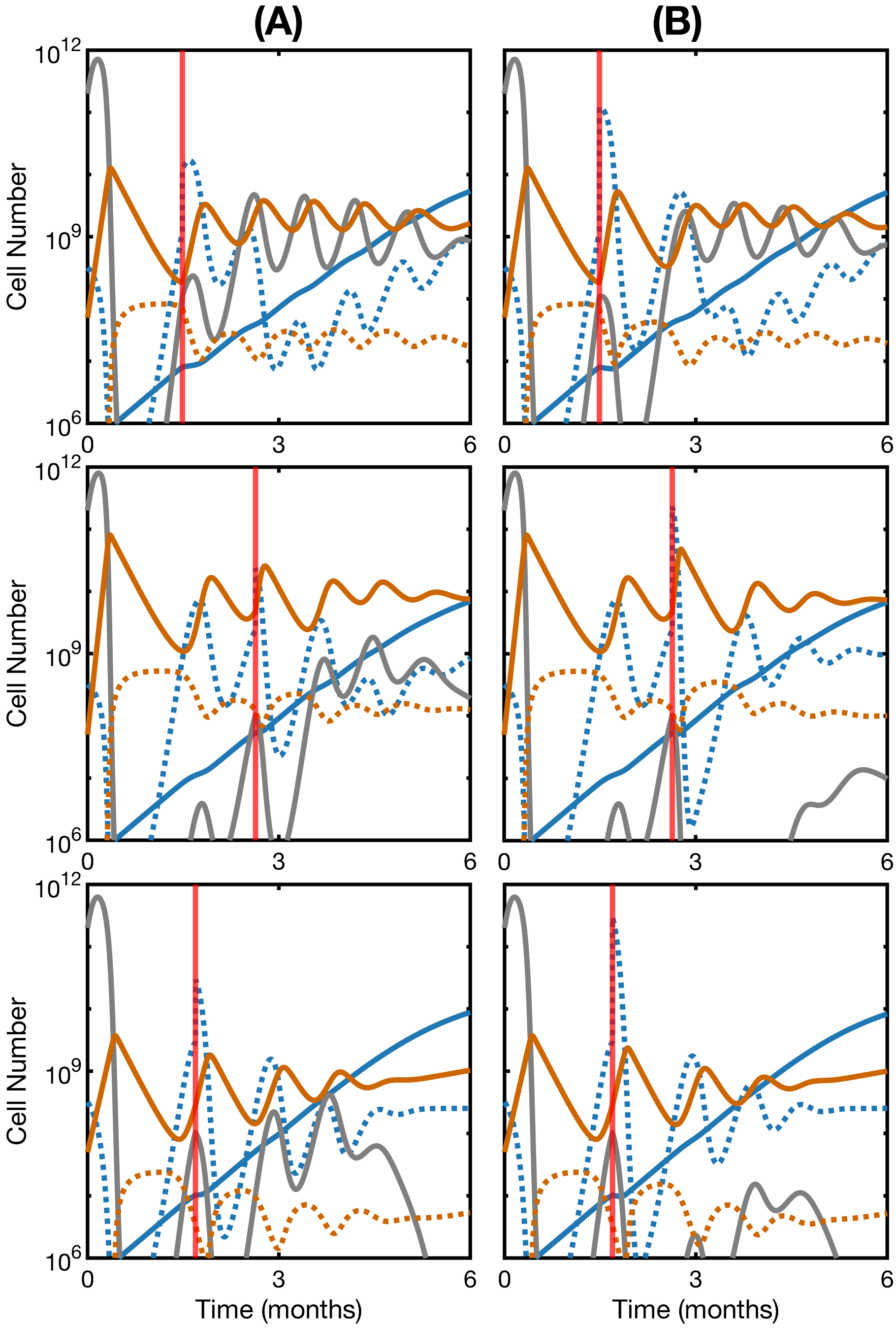

Figure S9: Effect of CD19+ B-cell infusion in non-responding patients. Dynamics of leukemic cells (solid gray line), CD19- and CD19+ B-cells (solid and dotted blue lines respectively) and activated and memory CAR T-cells (solid and dotted orange line, respectively), for the three non-responding patients of Figure 6. **(A)** Infusion of one order of magnitude of CD19+ B-cells at the time of relapse. **(B)** Infusion of two orders of magnitude of CD19+ B-cells at the time of relapse.

References

[1] Salvador Chulián, Álvaro Martínez-Rubio, Anna Marciniak-Czochra, Thomas Stiehl, Cristina Blázquez Goñi, Juan Francisco Rodríguez Gutiérrez, Manuel Ramírez Orellana, Ana Castillo Robleda, Víctor M Pérez-García, and María Rosa. Dynamical properties of feedback signalling in b lymphopoiesis: A mathematical modelling approach. *Journal of Theoretical Biology*, 522:110685, 2021.

[2] Howard E Skipper and Seymour Perry. Kinetics of normal and leukemic leukocyte populations and relevance to chemotherapy. *Cancer Research*, 30(6):1883–1897, 1970.

[3] Andreas Hirt, Eva-Maria Werren, Annette Ridolfi Luethy, Johannes Gerdes, and Hans P Wagner. Cell cycle analysis in lymphoid neoplasia of childhood: differences among immunologic subtypes and similarities in the proliferation of normal and leukaemic precursor b cells. *British journal of haematology*, 80(2):189–193, 1992.

[4] Jonathan Cooperman, Robert Neely, David T Teachey, Stephen Grupp, and John Kim Choi. Cell division rates of primary human precursor b cells in culture reflect in vivo rates. *Stem Cells*, 22(6):1111–1120, 2004.

[5] Helene Kraus, Sandra Kaiser, Konrad Aumann, Peter Bönelt, Ulrich Salzer, Dietmar Vestweber, Miriam Erlacher, Mirjam Kunze, Meike Burger, Kathrin Pieper, et al. A feeder-free differentiation system identifies autonomously proliferating b cell precursors in human bone marrow. *The Journal of Immunology*, 192(3):1044–1054, 2014.

[6] Abul K Abbas, Andrew H Lichtman, and Shiv Pillai. *Cellular and Molecular Immunology.* Elsevier Health Sciences, 2014.

[7] Sean C Bendall, Kara L Davis, El-ad David Amir, Michelle D Tadmor, Erin F Simonds, Tiffany J Chen, Daniel K Shenfeld, Garry P Nolan, and Dana Pe'er. Single-cell trajectory detection uncovers progression and regulatory coordination in human b cell development. *Cell*, 157(3):714–725, 2014.

[8] Emmanuelle Passegué, Amy J Wagers, Sylvie Giuriato, Wade C Anderson, and Irving L Weissman. Global analysis of proliferation and cell cycle gene expression in the regulation of hematopoietic stem and progenitor cell fates. *The Journal of experimental medicine*, 202(11):1599–1611, 2005.

[9] Rosana Pelayo, Kozo Miyazaki, Jiaxue Huang, Karla P Garrett, Dennis G Osmond, and Paul W Kincade. Cell cycle quiescence of early lymphoid progenitors in adult bone marrow. *Stem Cells*, 24(12):2703–2713, 2006.

[10] David Leitenberg, Joel M Rappeport, and Brian R Smith. B-cell precursor bone marrow reconstitution after bone marrow transplantation. *American journal of clinical pathology*, 102(2):231–236, 1994.

[11] JE Talmadge, E Reed, K Ino, A Kessinger, C Kuszynski, D Heimann, M Varney, J Jackson, JM Vose, and PJ Bierman. Rapid immunologic reconstitution following transplantation with mobilized peripheral blood stem cells as compared to bone marrow. *Bone marrow transplantation*, 19(2):161–172, 1997.

[12] P Lucio, A Parreira, MWM Van den Beemd, EG Van Lochem, ER Van Wering, E Baars, A Porwit-MacDonald, E Bjorklund, G Gaipa, A Biondi, et al. Flow cytometric analysis of normal b cell differentiation: a frame of reference for the detection of minimal residual disease in precursor-b-all. *Leukemia*, 13(3):419–427, 1999.

[13] Sergio Matarraz, Antonio Lopez, Susana Barrena, Carlos Fernández, Evan Jensen, J Flores, Paloma Bárcena, Ana Rasillo, José María Sayagués, ML Sánchez, et al. The immunophenotype of different immature, myeloid and b-cell lineage-committed cd34+ hematopoietic cells allows discrimination between normal/reactive and myelodysplastic syndrome precursors. *Leukemia*, 22(6):1175–1183, 2008.

[14] Cesar Nombela-Arrieta and Markus G Manz. Quantification and three-dimensional microanatomical organization of the bone marrow. *Blood advances*, 1(6):407–416, 2017.

[15] Andrew M Stein, Stephan A Grupp, John E Levine, Theodore W Laetsch, Michael A Pulsipher, Michael W Boyer, Keith J August, Bruce L Levine, Lori Tomassian, Sweta Shah, et al. Tisagenlecleucel model-based cellular kinetic analysis of chimeric antigen receptor–t cells. *CPT: pharmacometrics & systems pharmacology*, 8(5):285–295, 2019.

[16] Alexander A Ademokun and Deborah Dunn-Walters. Immune responses: primary and secondary. *eLS*, 2010.
